# Evaluation of Serological and Molecular Tests Used for the Identification of *Toxoplasma gondii* Infection in Patients Treated in an Ophthalmology Clinic of a Public Health Service in São Paulo State, Brazil

**DOI:** 10.3389/fcimb.2019.00472

**Published:** 2020-02-07

**Authors:** Fernando Henrique Antunes Murata, Mariana Previato, Fábio Batista Frederico, Amanda Pires Barbosa, Fabiana Nakashima, Geraldo Magela de Faria, Aparecida Perpétuo Silveira Carvalho, Cristina da Silva Meira Strejevitch, Vera Lucia Pereira-Chioccola, Lilian Castiglioni, Luiz Carlos de Mattos, Rubens Camargo Siqueira, Cinara Cássia Brandão de Mattos

**Affiliations:** ^1^Faculdade de Medicina de São José Do Rio Preto, São José Do Rio Preto, Brazil; ^2^FAMERP Toxoplasma Research Group, São José Do Rio Preto, Brazil; ^3^Ambulatório de Oftalmologia Do Hospital de Base, Fundação Faculdade Regional de Medicina de São José Do Rio Preto, São José Do Rio Preto, Brazil; ^4^Laboratório de Biologia Molecular de Parasitas e Fungos Do Centro de Parasitologia e Micologia, Instituto Adolfo Lutz, São Paulo, Brazil

**Keywords:** ocular toxoplasmosis, toxoplasma antibodies, *Toxoplasama gondii*, retinochoroiditis, polimerase chain reaction (PCR), qPCR, uveites

## Abstract

Ocular toxoplasmosis is one of the most common complications caused by the infection with the parasite *Toxoplasma gondii*. The risk of developing eye lesions and impaired vision is considered higher in Brazil than other countries. The clinical diagnosis is difficult and the use of sensitive and specific laboratorial methods can aid to the correct diagnosis of this infection. We compared serological methods ELISA and ELFA, and molecular cPCR, Nested PCR and qPCR for the diagnosis of *T. gondii* infection in groups of patients clinically evaluated with ocular diseases non-toxoplasma related (G1 = 185) and with lesions caused by toxoplasmosis (G2 = 164) in an Ophthalmology clinic in Brazil. Results were compared by the Kappa index, and sensitivity (S), specificity (E), positive predictive value (PPV), and negative (NPV) were calculated. Serologic methods were in agreement with ELISA more sensitive and ELFA more specific to characterize the acute and chronic infections while molecular methods were discrepant where qPCR presented higher sensitivity, however, lower specificity when compared to cPCR and Nested PCR.

## Introduction

Toxoplasmosis is a disease caused by the obligate intracellular parasite *Toxoplasma gondii*. In immunocompetent individuals the disease is usually asymptomatic, and its infection is commonly detected by serological tests (Saadatnia and Golkar, 2012). When symptomatic, ocular toxoplasmosis (OT) is the most common clinical manifestation (Garweg and Peyron, 2008; Tsirouki et al., 2018) which can be due to congenital or acquired infection (Montoya, 2002; Oréfice et al., 2010; Maenz et al., 2014).

The clinical manifestations result from tachyzoite invasion into host cells from an acute infection and also in chronic infection by the reactivation of tissue-cysts contained in the retina which release bradyzoites, leading to an intense inflammatory response and tissue destruction (Garweg and Peyron, 2008; Maenz et al., 2014; Tsirouki et al., 2018).

The prevalence of ocular toxoplasmosis in Brazil is high, and the severity and risk of ocular involvement are notably higher compared to the United States and Europe (Glasner et al., 1992; Garcia et al., 1999; Aleixo et al., 2009; Furtado et al., 2013; Grigg et al., 2015). Studies in the northwestern region of São Paulo showed that seroprevalence was 74.5%, of these, 27.3% had ocular disease (Ferreira et al., 2014).

The high rates of ocular disease caused by *T. gondii* infection in Brazil is still unknown, and it is still not clear why these strains can cause more ocular involvement than in the rest of the world. Genetic diversity of these strains and host immune response are important factors that have been related to the severity of this disease in Brazil (Grigg et al., 2001, 2015; Silveira et al., 2015; Greigert et al., 2019).

Clinical diagnosis is challenging and serological and molecular tests are mostly used to confirm the disease. However, there is still no consensus regarding which method would be the best to identify *T. gondii* infection (Garweg and Peyron, 2008; Maenz et al., 2014; Greigert et al., 2019).

Since there is no standard test for diagnosis of *T. gondii* infection in Brazil, the use of methods with higher sensitivity and specificity are essential to lead to the correct diagnosis of this disease. The aim of this study was to evaluate the serological and molecular methods for diagnosis of toxoplasmosis in patients with and without ocular lesions, suggestive of toxoplasmosis treated at the ambulatory of Ophthalmology at the Hospital de Base in the city of São José do Rio Preto, São Paulo, Brazil.

## Materials and Methods

### Ethics Statement

This study was approved by the Ethics Committee of the Medicine School in São José do Rio Preto (FAMERP-CAAE 32259714.8.0000.5415).

### Patients and Clinical Samples

This is a retrospective study that evaluated 349 blood samples from patients of both genders treated and clinically evaluated at the ambulatory of Ophthalmology of the Fundação Faculdade Regional de Medicina, Hospital de Base (FUNFARME), São José do Rio Preto, São Paulo, Brazil, from 2009 to 2014. All patients were invited to participate in the project, and signed the free and informed consent form after receiving all the information about the objectives and the procedures to be performed in this research. All selected patients were immunocompetent and were divided into two groups: Group 1 (G1): Patients with ocular injury caused by diseases such as glaucoma, diabetic retinopathy type I, retinal detachment, macular degeneration related to age, uveitis of unknown cause, corneal transplantation, cataract, macular changes, post-operative injury, among other eye diseases not related to toxoplasma infection (*N* = 185), and Group 2 (G2): Patients with uveitis characteristics of toxoplasmosis (*N* = 164). The criteria for inclusion in this group was the presence of lesions in the retina characteristics of toxoplasmosis and, retinochoroiditis with active lesions. Ocular clinical evaluation of all patients was performed by fundus examination, and photo documentation using fundus photography, angiography and OCT (Optical Coherence Tomography).

Peripheral blood samples were collected from all subjects in a dry tube for serological analysis and in a tube containing ethylenediaminetetraacetic acid (EDTA) for DNA extraction and molecular tests. Serological and molecular analyses were performed in the Immunogenetics Laboratory, Molecular Biology Department, FAMERP, São José do Rio Preto, São Paulo, Brazil.

### Serological Diagnosis

The presence of anti-*T. gondii* was confirmed using the semi-automated test by Enzyme Linked Immunosorbent assay (ELISA, DiaSorin, Italy) using the ETI-TOXOK-M reverse plus kit for IgM and ETI-TOXOK-G plus for IgG, and an automated test by enzyme linked fluorescent assay (ELFA, Biomerieux, France) using the Vidas^®^Toxo IgM kits (TXM) for IgM, Vidas^®^Toxo IgG II (TXG) to IgG and Vidas^®^Toxo IgG avidity (TXGA) for IgG avidity. The detection of IgM antibodies was performed by capture ELISA. The ELFA was performed in automated equipment (Mini Vidas, Biomerieux, France). Samples were considered positive for IgG antibodies by ELISA when the concentration was >15 IU/ml and negative when the IgG concentration was ≤ 15 IU/ml. For the IgM ELISA test, the absorbance values of the samples were compared with the average cut-off point, samples were considered positive when the absorbance values were higher than or equal to the cut-off limit point (>10% of the average cut-off) with the remaining samples being considered negative. Samples results with absorbance value between ±10% of the average cut-off were re-tested to confirm the result. By ELFA, samples were considered positive for IgG antibodies when >8 IU/mL, indeterminate from ≥4 to ≤ 8 IU/mL and negative when <4 IU/mL. For IgM antibodies, ELFA results were positive when the reagent index was ≥0.65 IU/mL, indeterminate from <0.65 to ≥0.55 IU/mL and negative <0.55 IU/mL. The IgG avidity was considered low when result was <0.200; intermediate avidity between ≤ 0.200 and <0.300; and high avidity when result was ≥0.300. The performance of the tests and results interpretation were made according to each manufacturer's instructions.

### Molecular Diagnosis

#### Genomic DNA Extraction

The genomic DNA was extracted from 5 ml of peripheral blood collected in EDTA tube using a commercial kit (Qiamp DNA blood mini kit, Qiagen, Germany) according to the protocol described by Mattos et al. (2011). The extracted DNA was stored at −20°C until the polymerase chain reaction (PCR) was performed.

#### Identification of *Toxoplasma gondii B1* Gene

##### Conventional polymerase chain reaction (cPCR)

Conventional PCR (cPCR) was performed to identify *T. gondii* DNA in blood samples. Two cPCR reactions were performed, one using the JW62/63 primer pair and the other using the B22/23 primer pair. The B22 (sense: 5′-AACGGGCGAGTAGCACCTGAGGAGA-3′) and B23 primers (anti-sense: 5′-TGGGTCTACGTCGATGGCATGACAACT-3′) amplify a 115 base-pair sequence of a specific repetitive region of the *B1* gene (accession numbers: *B1* gene *T. gondii* = GenBank: AF146527.1) (Burg et al., 1989; Colombo et al., 2005). The PCR mixture consisted of 8.5 μL of nuclease-free water (Promega, USA); 12.5 μL of GoTaq Green Master Mix (Promega, USA) and 1.0 μL of each B22 and B23 primers (25 pmol each—IDT, USA). DNA from patients and controls (5 μL in [100 ng/μL]) were added to the PCR mixture in a final volume of 25 μL. The PCR cycling conditions consisted of an initial denaturation step at 95°C for 5 min, 35 amplification cycles of 45 s at 95°C, 45 s at 62°C, and 45 s at 72°C with a final extension of 5 min at 72°C in a thermocycler (Verity, Applied Biosystems, USA). The PCR products were electrophoresed in 1.5% agarose gel using SYBR Safe stain (Invitrogen, USA).

##### Nested PCR

Conventional PCR was performed using the JW62 (antisense: 5′-TTCTCGCCTCATTTCTGGGTCTAC-3′) and JW63 primer pair (Sense: 5′-GCACCTTTCGGACCTCAACAACCG-3′), which amplifies a fragment of 288 base pairs of the *T. gondii B1* gene. The PCR mixture was prepared using 6.5 μL nuclease-free water (Promega, USA), 12.5 μL of GoTaq Green Master Mix (Promega, USA) and 0.5 μL of each of the JW62 and JW63 primers (10 μM each primer—IDT, USA). DNA from patients and controls (5 μL in [100 ng/μL]) were added to the PCR mixture in a final volume of 25 μL. The PCR cycling conditions consisted of an initial denaturation step at 95°C for 5 min, 40 amplification cycles of 45 s at 95°C, 45 s at 55°C, and 45 s at 72°C with a final extension of 5 min at 72°C in a thermocycler (Verity, Applied Biosystems, USA). The PCR products were electrophoresed in 1.5% agarose gel using SYBR Safe stain (Invitrogen, USA). The amplified product was subjected to a second PCR (Nested PCR) using the B22/23 primer pair following the protocol published by Okay et al. (2009) with modifications. The PCR mixture was prepared for the second reaction using 6.5 μL nuclease-free water (Promega, USA), 12.5 μL of GoTaq Green Master Mix (Promega, USA) and 0.5 μL of each of the B22 and B23 primers (25 pmol of each primer—IDT, USA). Five microliters from the first amplification reaction using the JW62/63 primer pair were added. The PCR cycling conditions consisted of an initial denaturation step at 95°C for 5 min, 25 amplification cycles of 45 s at 95°C, 45 s at 62°C, and 45 s at 72°C with a final extension of 5 min at 72°C in a thermocycler (Verity, Applied Biosystems, USA). The PCR products were electrophoresed in 1.5% agarose gel using SYBR Safe stain (Invitrogen, USA).

##### Real-time PCR (qPCR)

Genomic DNA was also subjected to real-time PCR (qPCR) using primers to amplify 16S rRNA gene. The primers used in the real-time PCR reactions were forward (5′-TGCATCCAACGAGTTTATAA-3′), reverse (5′-GGCATTCCTCGTTGAAGATT-3′), and TaqMan (FAM-ATTGCAATAATCTATCCCCATCACGATGCATAC-BBQ). Real-time PCR was performed in a Step One Plus system (Applied Biosystems, USA) using the following mixture: 4.5 μL nuclease-free water, 10.0 μL 2× QuantiTect Probe PCR Master Mix, 0.5 μL of PrimeTime kit (500 nM of each primer and 250 nM of probe) (Qiagen, Germany). DNA from patients and controls (5 μL in [100 ng/μL]) were added to the PCR mixture in a final volume of 25 μL. The PCR cycling conditions used for qPCR consisted of an initial denaturation step at 50°C for 2 min, once at 95°C for 15 min, 40 amplification cycles of 15 s at 94°C and 1 min at 60°C with a final extension of 30 s at 50°C. The primers and probe used in this analysis have been described by Gunel et al. (2012). Ultrapure water and DNA extracted from *T. gondii* (RH strain) were included as negative and positive controls, respectively in all PCR reactions (cPCR, Nested PCR and qPCR). To control the course of DNA extraction and check for PCR inhibitors, all samples were assayed using the HGH primer (Accession number: HGH = GenBank: U55206.1—sense: 5′-GCCTTCCCAACCATTCCCT-3′ and antisense: 5′-TCACGGATTTCTGTTGTGTTTC-3′), which amplifies a 400-base-pair fragment of the human growth hormone gene.

### Statistical Analysis

IBM SPSS software v.23 was used to determine the Kappa index (KI) and GraphPad Stat Software v. 3.06 to determine the sensitivity, specificity, positive predictive value and negative value. Sensitivities and specificities were calculated as: (i) percent of sensitivity = ratio of true positives/true positives + false negatives × 100; and (ii) percent of specificity = ratio of true negatives/true negatives +false positives × 100. *P* ≤ 0.05 was considered statistically significant. The strength of the agreement between two serological tests was calculated using the KI. The results are interpreted considering the ranges published by Landis and Koch (1977) where the agreement is considered poor, slight, fair, moderate, substantial and almost perfect when the KI is 0, 0–0.19, 0.2–0.39, 0.4–0.59, 0.6–0.79, and 0.8–1.0, respectively.

## Results

Group 1 (G1) was composed of 185 patients, 97 (52.4%) males and 88 (47.6%) females, with an average age of 51.6 years [range: 17–85; standard deviation (SD): 19.3]. G2 was composed of 164 patients, 95 (57.9%) males and 69 (42.1%) females, with an average age of 45.7 years (range: 10–90; *SD*: 19.6). The mean ages between G1 and G2 showed a statistically significant difference (*P* = 0.0054; student *t*-test = 2.799; *df* = 347; 95% confidence interval: 1,734–9,936).

In G1, serological tests detected 6 (IgM) and 121 (IgG) positive samples by ELISA, while 2 (IgM) and 119 (IgG) were positive by ELFA. For G2, 10 (IgM) and 158 (IgG) samples were positive by ELISA, while 6 (IgM) and 156 (IgG) samples were positive by ELFA. Compared results of serological tests are shown in Table 1.

**Table 1:** Comparison of serological and molecular tests of group without ocular toxoplasmosis (G1) with group with ocular toxoplasmosis (G2).

**Groups**	**Serological tests ELISA/ELFA positive**		**Molecular tests cPCR (JW62/63)/cPCR (B22/23)/Nested PCR/qPCR positive**
	**IgM**	**IgG**	
G1 (185 samples)	6 (3.2%)/2 (1.1%)	121 (65.4%)/119 (64.3%)	1 (0.5%)/1 (0.5%)/3 (1.6%)/3 (1.6%)
G2 (164 samples)	10 (6.1%)/6 (3.7%)	158 (96.3%)/156 (95.1%)	3 (1.8%)/10 (6.1%)/4 (2.4%)/14 (8.5%)

*Statistical analysis of IgG in G1. ELISA vs. ELFA: P-value = 0.913; 95% CI (0.875–1.181); IgM—ELISA vs. ELFA: P-value = 0.283; 95% CI (0.613–14.677); cPCR (JW62/63) vs. Nested PCR vs. cPCR (B22/23) vs. qPCR: P = 0.567; GL = 3; χ^2^ = 2.022. Statistical analysis of IgG in G2. ELISA vs. ELFA: P-value = 0.785; 95% CI (0.967–1.06); IgM—ELISA vs. ELFA: P-value = 0.443; 95% CI (0.619–4.481); cPCR (JW62/63) vs. Nested PCR vs. cPCR (B22/23) vs. qPCR: P = 0.012; GL = 3; χ^2^ = 10.936*.

*Statistical analysis of G1: cPCR (JW62/63) vs. Nested PCR vs. cPCR (B22/23) vs. qPCR: P = 0.567; GL = 3; χ^2^ = 2.022. Statistical analysis of G2: cPCR (JW62/63) vs. Nested PCR vs. cPCR (B22/23) vs. qPCR: P = 0.012; GL = 3; χ^2^ = 10.936*.

The KIs for the detection of anti-*T. gondii* IgG antibodies in G1 was 0.97 (almost perfect agreement between the two techniques, ELISA × ELFA), and 0.49 for IgM antibodies (moderate agreement between the two techniques, ELISA × ELFA). The KIs for anti-*T. gondii* antibodies of G2 was 0.85 (almost perfect agreement, ELISA × ELFA), and for IgM antibodies was 0.74 (substantial agreement between the two techniques, ELISA × ELFA).

Regarding molecular tests on G1, one sample was positive by one round-PCR using primer JW62/63 and by one round-PCR using B22/23. Nested-PCR using the primer B22/23 amplified three samples and qPCR using the 16S rRNA gene amplified three samples. On G2, one round-PCR using primer JW62/63 amplified three samples, and 10 by one round-PCR using B22/23. Nested-PCR using the primer B22/23 detected four samples and qPCR 16S rRNA gene amplified 14 samples. Results are shown in Table 1.

The sensitivity (S), specificity (E), positive predictive value (PPV), and negative (NPV) was calculated for each serological and molecular test separately. Results are presented in Table 2.

**Table 2:** Results for sensitivity (S), specificity (E), positive predictive value (PPV), and negative predictive value (NPV) between the serological tests in G1 and G2, performed by ELISA (DiaSorin) and ELFA (Biomerieux) and between the molecular tests performed by cPCR (JW62/63), Nested PCR, cPCR (B22/23), and qPCR.

	**S (%)**	**E (%)**	**PPV (%)**	**NPV (%)**
ELISA IgG	96.3	34.6	56.6	91.4
ELISA IgM	6.1	96.8	62.5	53.7
ELFA IgG	95.1	35.7	56.7	89.2
ELFA IgM	3.7	98.9	75.0	53.7
cPCR (JW62/63)	1.8	99.5	75.0	53.3
Nested PCR	2.4	99.5	80.0	53.5
cPCR (B22/23)	6.1	98.4	76.7	54.2
qPCR	8.5	98.4	82.3	54.8

## Discussion

This study evaluated serological and molecular methods used to identify *T. gondii* infection in patients treated at the Ophthalmology Clinic in the city of São José do Rio Preto, northwestern region of São Paulo state.

Most common enzyme immunoassays, ELISA and ELFA were evaluated in this study. ELISA detected more positive cases in both groups but for acute and chronic disease ELFA was more specific.

High sensitivity and specificity of serological test is essential, since a misdiagnosis would lead to wrong or late treatment of these patients, which could increase the changes of eye damage and loss of vision (Dhakal et al., 2015).

In this study, ELISA and ELFA had almost perfect agreement when compared by the Kappa index for the identification of IgG in both groups, indicating that these tests are very useful for the diagnosis of chronic infection. However, for IgM, Kappa index was moderate for G1 and with substantial agreement for G2 with higher detection by ELISA than ELFA.

All the samples tested positive for ELISA and negative for ELFA were also negative in the molecular tests, one sample was negative for IgG and high avidity of IgG for all samples. These findings may suggest that those IgM results detected by ELISA could be a result of persistence of Toxoplasma IgM in chronic infection. False positive results might be troublesome specially during prenatal care, as it could lead to undesirable consequences and unnecessary treatment and interventions, therefore, assays which do not detect these residual IgM antibodies would be ideal (Dhakal et al., 2015; Villard et al., 2016). Unfortunately, we just had access to one sample of these patients and consequently no follow-up was performed. In any case, confirming the IgM test is not easy since there is no reference method for its detection (Dhakal et al., 2015). The use of a test that could eliminate the risks of detecting residual IgM would be paramount, since a follow-up study to confirm the infection is expensive and time-consuming (Gras et al., 2004).

Automated method as ELFA have shown high sensitivity and specificity when compared to other methods with advantages of eliminating interferences that may occur during manual testing (Del Bono et al., 1989; Murat et al., 2013). The evaluation of IgM antibodies in the acute infection has been discussed since it still can be detected in chronic infection and there is a risk of false-positive results by cross-reactivity with antibodies, rheumatoid factor and other viral and bacterial diseases (Naot et al., 1981; Montoya, 2002; Bichara et al., 2012; Villard et al., 2012). In a study conducted by Dao et al. (2003) comparing the reaction of IgM antibodies by ELISA and ISAGA in patients without clinical suspicion of infection by *T. gondii*, it was observed that 5 samples were positive in ELISA but none in ISAGA. The authors concluded that the different antigen compositions in solid phase reactions may have led to false-positive results by ELISA (Dao et al., 2003). The difference in the composition of the antigens of ELFA and ELISA kits may also have contributed to the difference finding in our study.

The low specificity of the IgG in this study could be related to high rates of seroprevalence in the region, and the permanence of these antibodies for the whole life of the host, even without the clinical signs of the disease. Melamed describes the difficulty of serologic diagnosis in patients with eye injuries, as these antibodies are present in patients with or without clinical signs of the disease, making the proper identification of the etiologic agent difficult (Melamed, 2009).

Since there is no standardization to detect *T. gondii* by PCR, different protocols have been used (Roux et al., 2018; Greigert et al., 2019). Selection of primer, applied technology and a more suitable sample are some reasons for this challenge (Saadatnia and Golkar, 2012). Several studies analyzing different targets and samples were done and there is still no consensus of the best test (Homan et al., 2000; Jones et al., 2000; Calderaro et al., 2006; Okay et al., 2009; Menotti et al., 2010).

In a study conducted by Jones et al. (2000) comparing three *T. gondii* genes (*B1*, P30, and 16S rRNA gene) in aqueous humor, *B1* was more sensitive than P30 and 16S rRNA gene, when it was submitted to a nested-PCR. In our study, 16S rRNA gene was more sensitive than *B1* and less specific when compared to one-round PCR with JW62/63 and nested-PCR, and same specificity compared with one-round on *B1* conventional PCR. Some factors may have contributed for these results.

First, 16S rRNA gene is the most highly repeated region of the gene studied (110 copies in the *T. gondii* genome) compared to 35 copies of *B1* gene, increasing the chances for amplification (Jones et al., 2000; Calderaro et al., 2006; Ivovic et al., 2012).

Second, the kind of specimen analyzed, as it seems that results of molecular tests can vary according to the kind of sampling, as shown by Calderaro et al. (2006) who found same sensitivity between nested-PCR using *B1* gene and real time PCR using 16S rRNA gene when analyzing blood samples and less sensitivity of 16S rRNA gene when analyzing cerebrospinal fluid samples.

The sensitivity of B1 gene was higher when samples were submitted just to one-round PCR using B22/23 primer than compared to one-round PCR using JW62/63 and nested-PCR. Primer B22/23 amplifies a 115-base pair sequence of *B1* gene and has been reported as highly sensitive and specific primer used to detect *T. gondii* DNA in blood, cerebrospinal fluid, and amniotic fluid (Vidal et al., 2004; Okay et al., 2009; Mattos et al., 2011; Camilo et al., 2017; Murata et al., 2017). In a study conducted by Camilo et al. (2017) evaluating two real time-PCR for *B1* gene and REP-529 with a conventional PCR using the B22/23 primer, the authors found that REP-529 had better performance compared with the *B1* gene. However, the primer B22/23 had the same rate of detection as REP-529 (Camilo et al., 2017).

The lowest detection of *T. gondii* DNA was observed when samples were submitted to a cPCR using primers JW62/63 and nested-PCR. Contrary to our results, Okay et al. (2009) found more positive results when analyzed amniotic fluid samples using the JW62/63 (120/467) than using the 16S rRNA gene (0/467). The authors also submitted 50 samples from negative result on JW62/63 to a nested-PCR using primer B22/23, which detected more nine positive samples (Okay et al., 2009). In our study, all samples analyzed with JW62/63 were also submitted to a nested-PCR using the primer B22/23 irrespectively to the first one-round result. All the samples positive on the JW62/63 were also positive for the nested-PCR, which detected three more positive samples, suggesting that nested-PCR can be more sensitive than conventional PCR (Jones et al., 2000; Okay et al., 2009).

This study shows that the most common used serological tests ELISA and ELFA are good tests for the detection of *T. gondii* antibodies in the groups of patients analyzed with higher sensitivity for ELISA but better specificity for ELFA. For molecular tests, real time PCR using the 16S rRNA gene was the most sensitive, however, less specific than JW62/63 and nested-PCR using the primer B22/23.

Despite the limitation of this study related to the lack of follow up of these patients, our results show that even with no consensus of the best protocol to use, the combinate use of these tests with clinical evaluation and follow up could be a great tool for the correct diagnosis of *T. gondii* infection.

## Data Availability Statement

All datasets generated for this study are included in the article/supplementary material.

## Ethics Statement

The studies involving human participants were reviewed and approved by the Ethics Committee of the Medicine School in São José do Rio Preto (FAMERP-CAAE 32259714.8.0000.5415). The patients/participants provided their written informed consent to participate in this study.

## Author Contributions

CB and FM coordinated the experiments and designed the study. CB, FM, LM, and VP-C wrote the manuscript. MP, FF, RS, and AB performed the selection of clinical samples and clinical evaluation. FM, MP, FN, AS, GF, CM, and VP-C performed the serological and molecular diagnosis for toxoplasmosis. FM and LC performed the statistical analyses. All authors contributed substantially to the interpretation of the data and to the manuscript. In addition, all authors revised the manuscript, approved the final version submitted, published, and agreed to be accountable for all aspects of the work in ensuring that questions related to the accuracy or integrity of any part of the work are appropriately investigated and resolved.

### Conflict of Interest

The authors declare that the research was conducted in the absence of any commercial or financial relationships that could be construed as a potential conflict of interest.
